# Effects of dietary lipids on the hepatopancreas transcriptome of Chinese mitten crab (*Eriocheir sinensis*)

**DOI:** 10.1371/journal.pone.0182087

**Published:** 2017-07-28

**Authors:** Banghong Wei, Zhigang Yang, Jianyi Wang, Aqin Chen, Qiuyan Shi, Yongxu Cheng

**Affiliations:** College of Fisheries and Life Science, Shanghai Ocean University, Shanghai, China; Institute of Oceanology, Chinese Academy of Sciences, CHINA

## Abstract

Fish oil supplies worldwide have declined sharply over the years. To reduce the use of fish oil in aquaculture, many studies have explored the effects of fish oil substitutions on aquatic animals. To illustrate the effects of dietary lipids on Chinese mitten crab and to improve the use of vegetable oils in the diet of the crabs, 60 male juvenile Chinese mitten crabs were fed one of five diets for 116 days: fish oil (FO), soybean oil (SO), linseed oil (LO), FO + SO (1:1, FSO), and FO + LO (1:1, FLO). Changes in the crab hepatopancreas transcriptome were analyzed using RNA sequencing. There were a total 55,167 unigenes obtained from the transcriptome, of which the expression of 3030 was significantly altered in the FLO vs. FO groups, but the expression of only 412 unigenes was altered in the FSO vs. FO groups. The diets significantly altered the expression of many enzymes involved in lipid metabolism, such as pancreatic lipase, long-chain acyl-CoA synthetases, carnitine palmitoyltransferase I, acetyl-CoA carboxylase, fatty acid synthase, and fatty acyl Δ9-desaturase. The dietary lipids also affected the Toll-like receptor and Janus activated kinase-signal transducers and activators of transcription signaling pathways. Our results indicate that substituting fish oil with vegetable oils in the diet of Chinese mitten crabs might decrease the digestion and absorption of dietary lipids, fatty acids biosynthesis, and immunologic viral defense, and increase β-oxidation by altering the expression of the relevant genes. Our results lay the foundation for further understanding of lipid nutrition in Chinese mitten crab.

## Introduction

As a source of energy, essential fatty acids, phospholipids, and some fat-soluble vitamins, lipids are indispensable in aquatic feed, particularly for crustaceans [1, 2]. Over the years, fish oil (FO) produced by wild fisheries has been the main lipid resource in aquatic feed because of the unidentified growth factors and high content of n-3 highly unsaturated fatty acids (HUFAs), such as eicosapentaenoic acid (EPA, 20:5n-3) and docosahexaenoic acid (DHA, 22:6n-3) [3]. However, given the development of aquaculture and the decline of wild fisheries, FO cannot meet the demands of aquaculture industry, and has become an obstacle to the development of the aquaculture industry. For the sustainable development of the aquaculture industry, many studies have been conducted to identify other lipid resources to substitute FO in fish and crustacean diets [4–7]. Some vegetable oils, such as rapeseed, linseed, and soybean oil, are considered good alternative lipid resources that are cheap and easily obtained [8]. However, many studies have indicated that the use of vegetable oils is limited by the anti-nutritional factors, poor palatability, and insufficient levels of essential fatty acids [9, 10]. On the other hand, vegetable oils could partially replace FO without affecting the growth performance and feed conversion in many fish, but the fatty acid composition in the liver and muscle are closely related to diet [11, 12]. The same results have been reported in some crustaceans, and vegetable oils might even be better for crustacean growth [5, 13], but little is known about the molecular mechanism of FO substitution. Therefore, the effects of substituting FO in crustaceans warrant further study.

The Chinese mitten crab (*Eriocheir sinensis*) is a native species in East Asia, and has become the most important economic crab species in China [14]. The maximum growth of most crustaceans can be induced by 2–10% of total lipids of the diet (dry weight) [15]. Most crustaceans prefer shorter chain and saturated fatty acids for energy [16]; however, polyunsaturated fatty acids (PUFAs) also play an important role in many crustacean physiological functions, for example, arachidonic acid (ARA, 20:4n-6), EPA, and DHA are closely related to molting [17] and can improve growth and immunity in the early growth stages of *Litopenaeus vannamei* [9]. Previously, we had researched lipid nutrition of *E*. *sinensis*. Most of our results suggested that substituting FO in the diet of *E*. *sinensis* is practicable, where vegetable oil could partially replace FO without affecting growth, but the fatty acid composition could be significantly altered [18–20]. To increase FO substitution, the mechanism of the effects of substituting FO should be investigated.

A next-generation sequencing technique, RNA sequencing (RNA-Seq), is a newly developed technology used for studying molecular mechanisms in biological studies [21], and has been successfully used for studying *E*. *sinensis*. However, most studies have focused on *E*. *sinensis* development, molting, immune pathways, relationships between nutrition and reproduction, osmoregulation, and adaptation to eyestalk ablation [22–27]. Few researchers have investigated the effects of dietary lipid resources on *E*. *sinensis*. In this study, two vegetable oils, which mainly contain ω-3 and ω-6 fatty acids respectively, were selected as the substitution of fish oil in the diets of *E*. *sinensis*. The ratio of the replacement was determined according to our results before. To illustrate the mechanism of the replacement of fish oil, two groups with the complete replacement of fish oil were added to enlarge the effects of the replacement. Then we analyzed the hepatopancreas transcriptome of *E*. *sinensis* fed with different diets, and determined the effects of different dietary lipids on the lipid metabolism in *E*. *sinensis*.

## Materials and methods

### Experimental diets

Five isonitrogenous, isolipidic purified diets were formulated from three lipid resources: FO, soybean oil (SO), and linseed oil (LO). Table 1 lists the ingredients of the experimental diets. The diets were formed into 1.5-mm (diameter) pellets and stored at -20°C until used.

**Table 1:** Composition of the experimental diets.

Ingredients	Fish oil	Soybean oil	Linseed oil	Fish oil and soybean oil	Fish oil and linseed oil
	FO	SO	LO	FSO	FLO
Casein	41	41	41	41	41
Cellulose	4	4	4	4	4
Wheat flour	28.65	28.65	28.65	28.65	28.65
Carboxymethylcellulose (CMC)	4	4	4	4	4
Yeast extract	5	5	5	5	5
Lysine	0.15	0.15	0.15	0.15	0.15
Glycine	0.5	0.5	0.5	0.5	0.5
Vitamin C (99.7%)	0.5	0.5	0.5	0.5	0.5
Vitamin E (97%)	0.1	0.1	0.1	0.1	0.1
Phospholipid (99%)	3	3	3	3	3
Cholesterol	0.5	0.5	0.5	0.5	0.5
Inositol	0.6	0.6	0.6	0.6	0.6
Choline chloride (50%)	1	1	1	1	1
Mineral premix^1^	3	3	3	3	3
Vitamin premix^2^	2	2	2	2	2
Fish oil	6	0	0	3	3
Soybean oil	0	6	0	3	0
Linseed oil	0	0	6	0	3
Proximate composition (percentage of dry weight)					
Crude protein	39.26	39.24	39.44	39.80	39.46
Crude lipid	9.75	9.31	9.47	9.82	9.56
Ash	5.68	5.77	5.72	5.74	5.70

^1^Mineral premix: 1 kg diet contained Ca(H_2_PO_4_)_2_, 10 g; MgSO_4_⋅7H_2_O, 2.4 g; KCl, 4.5 g; NaCl, 2.1 g; FeSO_4_⋅H_2_O, 155 mg; CuSO_4_⋅5H_2_O, 40 mg; ZnSO_4_⋅H_2_O, 80 mg; MnSO_4_⋅H_2_O, 30 mg; KI, 11.7 mg; CoCl_2_⋅6H_2_O, 4.8 mg; Na_2_SeO_3_, 2.4 mg.

^2^Vitamin premix: 1 kg diet contained vitamin A, 10000 IU; vitamin D, 2500 IU; vitamin K, 64 mg; thiamin, 60 mg; riboflavin, 250 mg; pyridoxine, 60 mg; calcium pantothenate, 240 mg; niacin, 60 mg; folic acid, 12 mg; biotin, 50 mg; cyanocobalamin, 4 mg.

### Experimental animals and feeding trials

Juvenile Chinese mitten crabs were obtained from the Chongming research base of Shanghai Ocean University and were stocked in tanks for 1 week for acclimation. During this period, the crabs were fed FO diet. After 1 week, 60 healthy male crabs (initial weight 2.15 ± 0.10 g) were randomly assigned to five groups (n = 12). Each crab in each group was cultivated in a single plastic box (36 cm × 18 cm × 18 cm). The groups were randomly assigned one experimental diet and were fed once daily at 13:00 h for 116 days. Uneaten feed was removed with a siphon tube after 2 h. During the experiment, the water was exchanged once daily with 1/3–1/2 of the tank volume, and was aerated throughout the feeding trial. The photoperiod was approximately 12-h light:12-h dark. Water quality parameters were monitored 2–3 times weekly to maintain conditions of 24.5–30.0°C, pH 8.0 ± 0.4, dissolved oxygen > 5 mg/L, and total ammonia nitrogen < 0.01 mg/L.

At the end of the experiment, the crabs were fasted for 24 h. Three crabs were randomly collected from each group, and were dissected to obtain the hepatopancreas for transcriptome analysis. Then the hepatopancreas was immediately frozen in liquid nitrogen and stored at -80°C until used.

### RNA extraction, transcriptome library preparation, and RNA-Seq

Total RNA was extracted from the hepatopancreas using TRIzol (Invitrogen) according to the manufacturer’s instructions. RNA quality and quantity were determined using Agilent 2100 and NanoDrop 2000 prior to subsequent experiments. Only high-quality RNA samples (1.8 < optical density [OD]_260/280_ < 2.2; 28S:18S > 1.0; RNA >5 μg) was used for the transcriptome analysis.

The RNA-Seq transcriptome library was prepared using a Truseq RNA Sample Prep Kit (Illumina). PolyA mRNA was purified using poly-T oligo attached magnetic beads (Invitrogen), and was randomly segmented into 200-bp fragments by fragmentation buffer. Then, first-strand complementary DNA (cDNA) was synthesized using reverse transcriptase and random primers, followed by synthesis of second-strand cDNA. The second-strand cDNA was end-repaired using End Repair Mix (Illumina), and a single A base was added at the 3ʹ end for adapter ligation. The cDNA target fragments were selected on 2% Low Range Ultra Agarose (Bio-Red), followed by 15 cycles of PCR amplification. After TBS-380 (Invitrogen) quantification, bridge PCR was performed to amplify the DNA fragments to single-molecule DNA clusters, which were subsequently used in HiSeq 4000 (Illumina) sequencing.

### De novo assembly and annotation

After quality trimming and adapter clipping by SeqPrep (https://github.com/jstjohn/SeqPrep) and Sickle (https://github.com/najoshi/sickle), clean data were obtained for RNA de novo assembly with Trinity (http://trinityrnaseq.sourceforge.net/, Version: trinityrnaseq-r20140413) [28]. For annotation, the assembled transcripts were aligned with the NCBI protein nonredundant (Nr), STRING, Swiss-Prot, and Kyoto Encyclopedia of Genes and Genomes (KEGG) databases using BlastX (Version 2.2.25), with a cut-off E-value < 1.0 × 10^−5^. Gene Ontology (GO) functional classification was conducted to obtain GO annotations for describing biological processes, molecular functions, and cellular components using Blast2GO (http://www.blast2go.com/b2ghome) [29]. The KEGG (http://www.genome.jp/kegg/) was used to analyze the pathways in which the transcripts were involved.

### Differential gene expression and functional enrichment

Expression abundance was determined using RSEM (http://deweylab.biostat.wisc.edu/rsem/) [30]. Read counts were obtained by mapping each sample to the corresponding gene. The gene expression levels were measured according to the fragments per kilobase of exon model per million mapped reads (FPKM) method. Differential expression analysis was performed using edgeR (http://www.bioconductor.org/packages/2.12/bioc/html/edgeR.html). Genes were considered significantly differentially expressed (DEGs) when the false discovery rate < 0.05 or log_2_|FC| ≥ 1. Cluster analysis was performed according to the DEG expression levels. GO and KEGG pathway functional enrichment analyses were then carried out on the DEGs to determine the DEG functions. Goatools (https://github.com/tanghaibao/goatools) and KOBAS (http://kobas.cbi.pku.edu.cn/home.do) were used for GO and KEGG pathway functional enrichment analysis, respectively. Hypergeometric distribution was used to obtain the P-value; significant enrichment was regarded as corrected P-value < 0.05.

### Quantitative real-time PCR validation of RNA-Seq

Ten DEGs were randomly selected for validation by quantitative real-time PCR (qRT-PCR); Table 2 lists the primer sequences. Gene expression was normalized to β-actin. Total RNA was extracted from the hepatopancreas of three crabs in each group using TRIzol (Invitrogen) according the manufacturer’s instructions. Then, first-strand cDNA was synthesized using PrimeScript RT Master Mix (Cat. No. RR036A, TaKaRa). The qRT-PCR was carried out following the manufacturer’s instructions for SYBR Premix Ex Taq (Cat. No. RR420A, TaKaRa) in an ABI 7500 Real-Time PCR System (Life Tech, applied biosystems) using the template above. Each sample was triplicate to reduce the error caused by the PCR system. The qRT-PCR was carried out in a total volume of 10 μL: 5 μL 2× SYBR Premix Ex Taq, 0.2 μL 50× ROX Reference Dye II, 1 μL diluted cDNA mix, 0.2 μL each primer (10 μM), and 3.4 μL sterile distilled water. The transcript levels were calculated using the comparative threshold cycle (2^-ΔΔCt^) formula. ΔCt was obtained in the formula: ΔCt = Ct gene of interest—Ct internal control, then a maximum ΔCt was selected as ΔCt_max_, ΔΔCt was calculated in the formula: ΔΔCt = ΔCt−ΔCt_max_. Then the relative expressions of each genes were defined by 2^-ΔΔCt^, more information about the formulation of the comparative threshold cycle (2^-ΔΔCt^) formula was referred to Schmittgen [31]. After log-transformation, FPKM value of each group in RNA-seq were compared to the results of qRT-PCR for the validation of RNA-Seq.

**Table 2:** Primers used for qRT-PCR.

Gene	Sense Primer	Anti-Sense Primer
GAPDH	TCGGTATCAACGGATTCGG	GGGGTCATTCACAGCCACAA
TLSP	AGACACATAGGCCCATCCCA	TTCACACTGCCCCAACACTC
FAD9	TAAGGTGGTGTGGAGAAACG	ATCAGGGTGAAGCCTAGGGT
ALF3	GGGATGGCGGAGTGTAACAA	GACAGGAAGGAAAACATGAGGT
PPAF	CAGGTGTGGATAAAGTTGGG	GTATGAAAAGTGTAGGGGCG
HPGDS	GAGGACATCACCCCAAAGC	TCATGGTCAGCCCGAGAG
PL	CACCACCTTCTCCCTCTT	CGTGCACCACTACCTTGA
CC	CGACTGGATGATGTCACCAA	GTATCTACGACGCCATTGCT
FABP	ATCACCAGTCCCACACCCAA	CGCACCTCAACTCCACTACAAT
LGBP	GACATTGTGGAATGCAGGG	TGAGTCAACGAAGTCGGAGG
β-Actin	ACCTCGGTTCTATTTTGTCGG	ATGCTTTCGCAGTAGTTCGTC

GAPDH: Glyceraldehyde-3-phosphate dehydrogenase; TLSP: Trypsin-like serine protease; FAD9: Acyl-CoA Δ9-desaturase; ALF3: Anti-lipopolysaccharide factor 3; PPAF: Prophenoloxidase-activating factor; HPGDS: Hematopoietic prostaglandin D synthase; PL: Pancreatic lipase; CC: Cryptocyanin; FABP: Fatty acid-binding protein; LGBP: Lipopolysaccharide and β-1,3-glucan-binding protein.

## Results

### Sequencing and de novo assembly

After sequencing, quality trimming, and adapter clipping, a total 320,973,688 reads were obtained from the hepatopancreas of *E*. *sinensis* fed FO, SO, LO, FSO (FO + SO), or FLO (FO + LO) diet (Table 3) and used for de novo assembly. We obtained 70,591 transcripts after assembly, and the transcripts were further clustered into 55,167 unigenes. The average transcript and unigene length was 946 bp and 1083 bp, respectively. Table 4 shows the other statistics of the assembly. About 22,760 transcripts (32.24%) and 20,929 unigenes (37.94%) were 1–400 bp in length, accounting for the majority of the transcripts and unigenes.

**Table 3:** Summary of the RNA-Seq reads production after quality trimming and adapter clipping.

Sample	Reads	Nucleotides	Q20%	Q30%	GC%
FO	67921784	9965463329	98.7	96.05	51.82
SO	63379752	9295537814	98.73	96.17	49.34
LO	58608878	8590472597	98.67	96	47.8
FSO	58790294	8630748381	98.72	96.12	51.45
FLO	72272980	10595523301	98.71	96.1	49.1

Q20%, Q30%: percent of bases with Phred value > 20 and >30.

GC%: percent of G and C bases accounting for total bases.

**Table 4:** Summary of RNA-Seq de novo assembly results.

	Unigenes	Transcripts
Total sequence number	55167	70591
Total sequence bases	52192785	76469410
Percent of GC (%)	47.64	47.83
Longest transcript bases	18906	18906
Shortest transcript bases	201	201
Average length bases	946	1083

### Annotation of unigenes

The assembled unigenes were aligned with the Nr, STRING, Swiss-Prot, and KEGG databases using BlastX. Of the assembled unigenes, 25,920 (46.98%), 17,499 (31.72%), and 14,532 (26.34%) were matched in the Nr, Swiss-Prot, and KEGG databases, respectively; only 5820 (10.55%) were matched in the STRING database. Up to 13,305 unigenes were matched in the Nr database with 0 < E-value ≤ 1 × 10^−10^.

GO annotation analysis showed that the unigenes could be assigned to three parts: biological process, cellular component, and molecular function, and could be further classified into 62 categories (S1 Fig). Most of the unigenes were in the terms cellular process, single-organism process, metabolic process, cell, cell part, binding, and catalytic activity. The Clusters of Orthologous Groups (COG) number was obtained from the results of the blast with the STRING database, and the unigenes were classified under their function according to the COG number. The most enriched terms were in general function prediction only, followed by post-translational modification, protein turnover, chaperones and translation, ribosomal structure, and biogenesis (S2 Fig). According to the KEGG pathway, in which the unigenes participated, the unigenes were assigned to five processes: metabolism (46.2%), genetic information processing (13.9%), environmental information processing (11.0%), cellular process (10.4%), and organismal systems (18.5%) (S3 Fig). In metabolism, global and overview maps was the most highly represented, followed by amino acid metabolism, carbohydrate metabolism, energy metabolism, and lipid metabolism. Translation, signal transduction, transport and catabolism, endocrine system comprised the main portion of the remaining four processes, respectively.

### Analysis of DEGs

**Fig 1:**
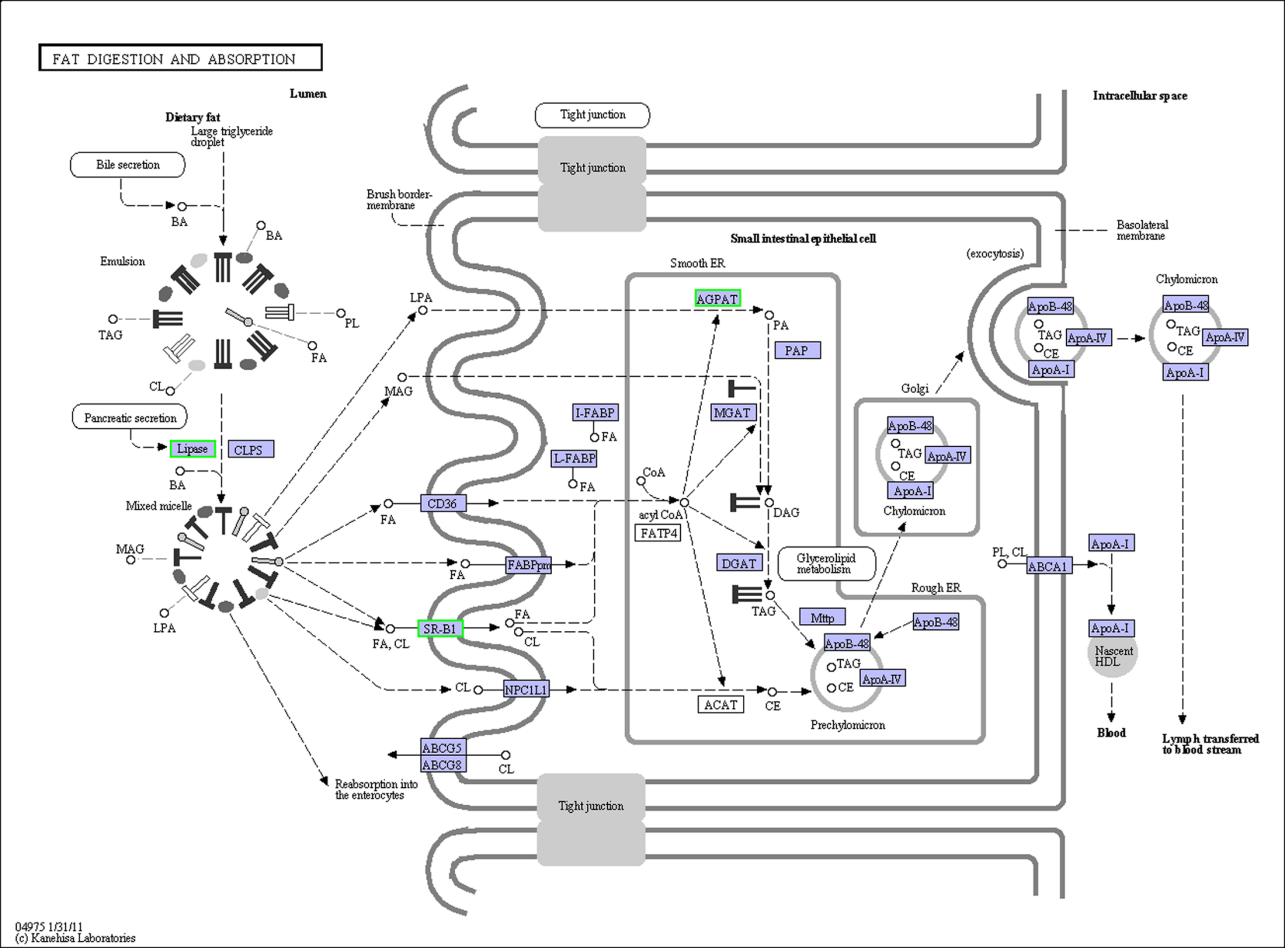
Fat digestion and absorption pathway.

**Fig 2:**
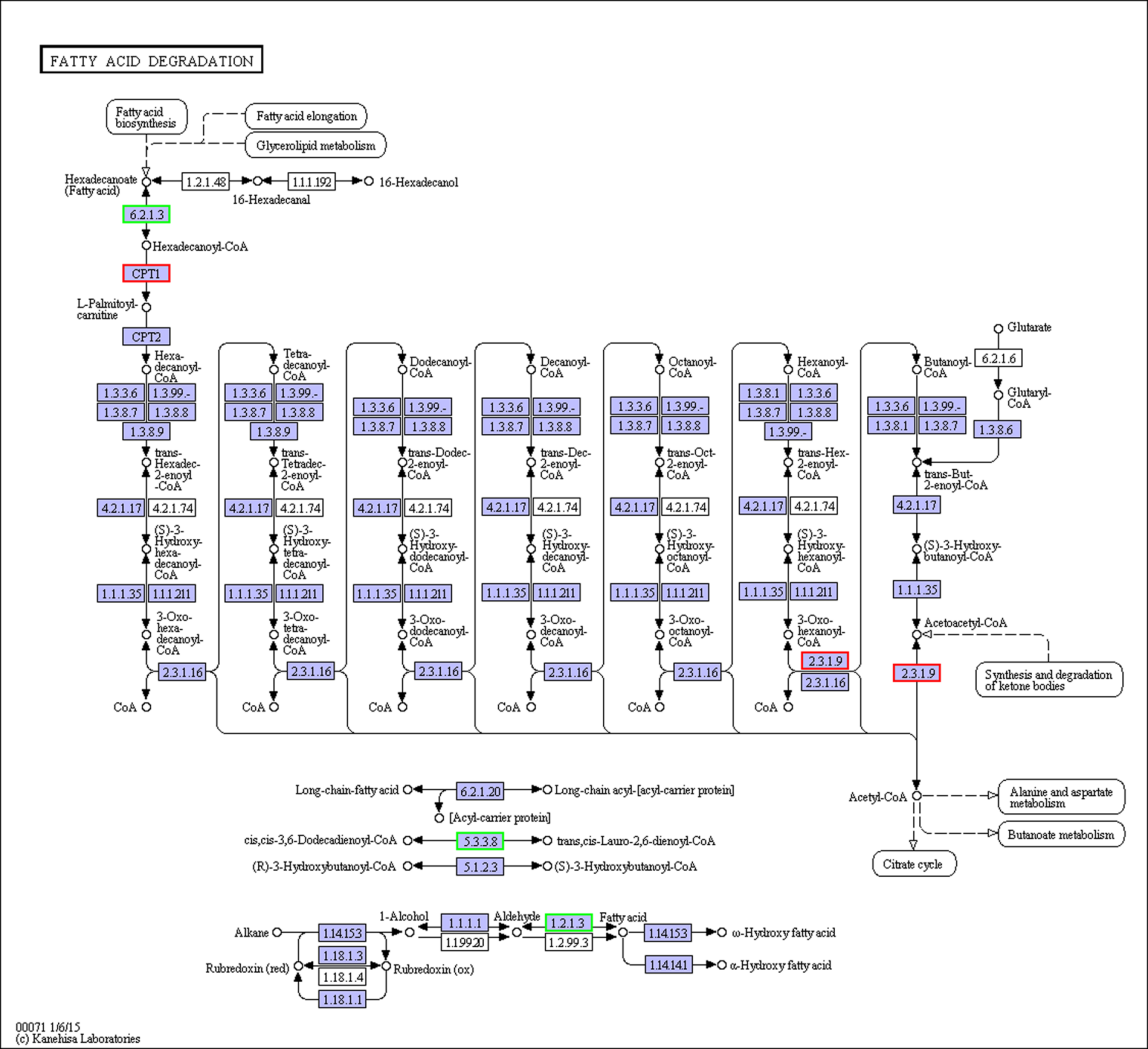
Fatty acid degradation pathway.

**Fig 3:**
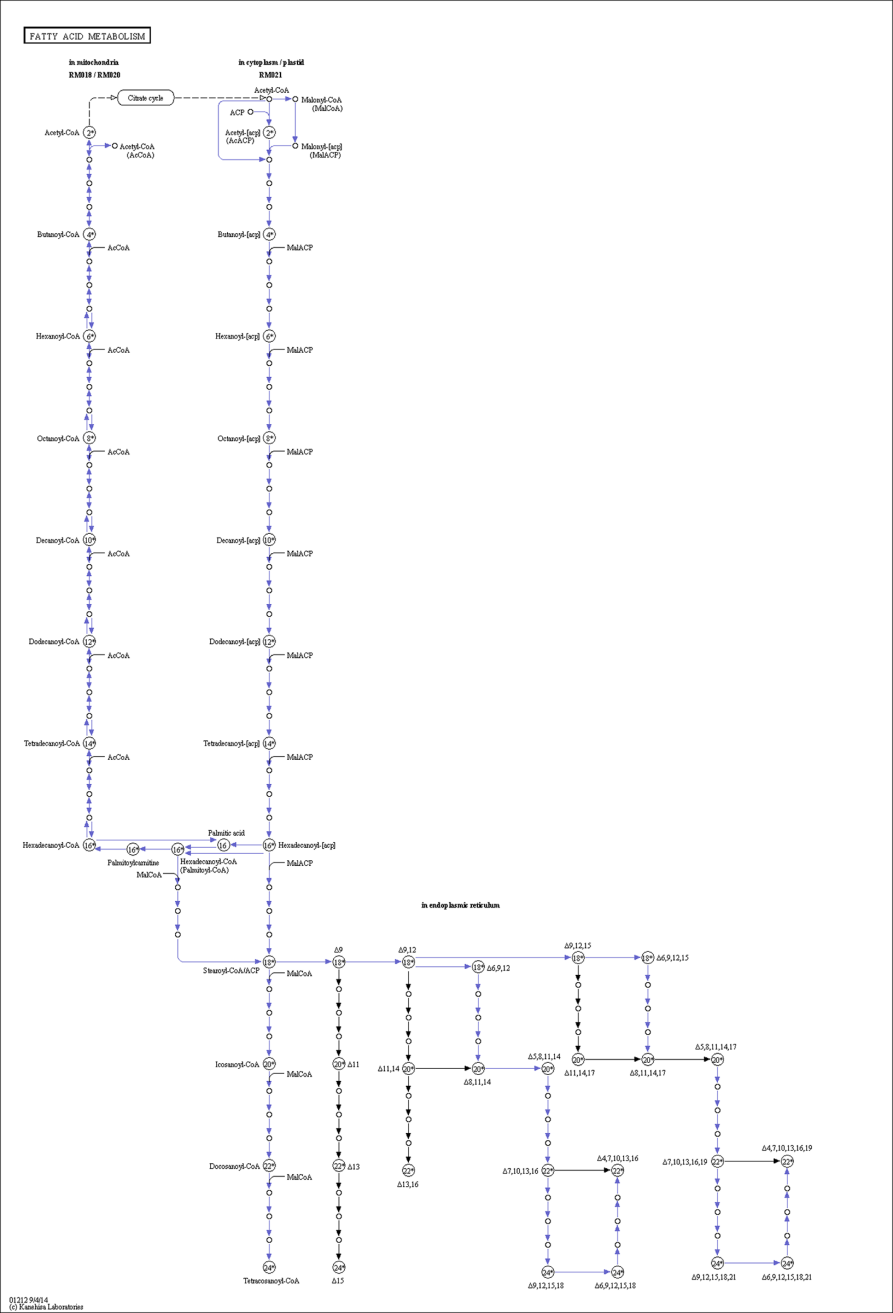
Fatty acid metabolism pathway.

**Fig 4:**
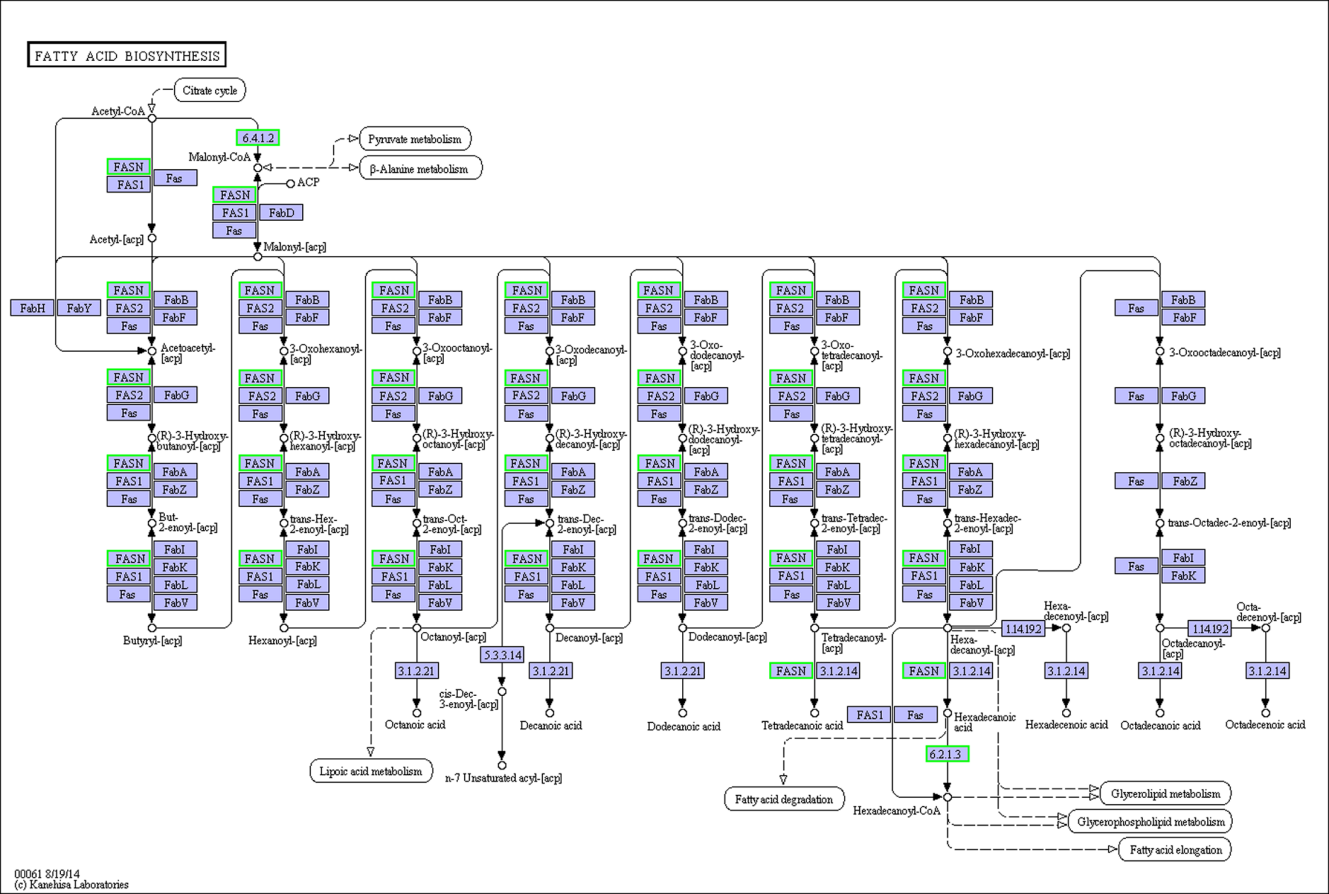
Fatty acid biosynthesis pathway.

**Fig 5:**
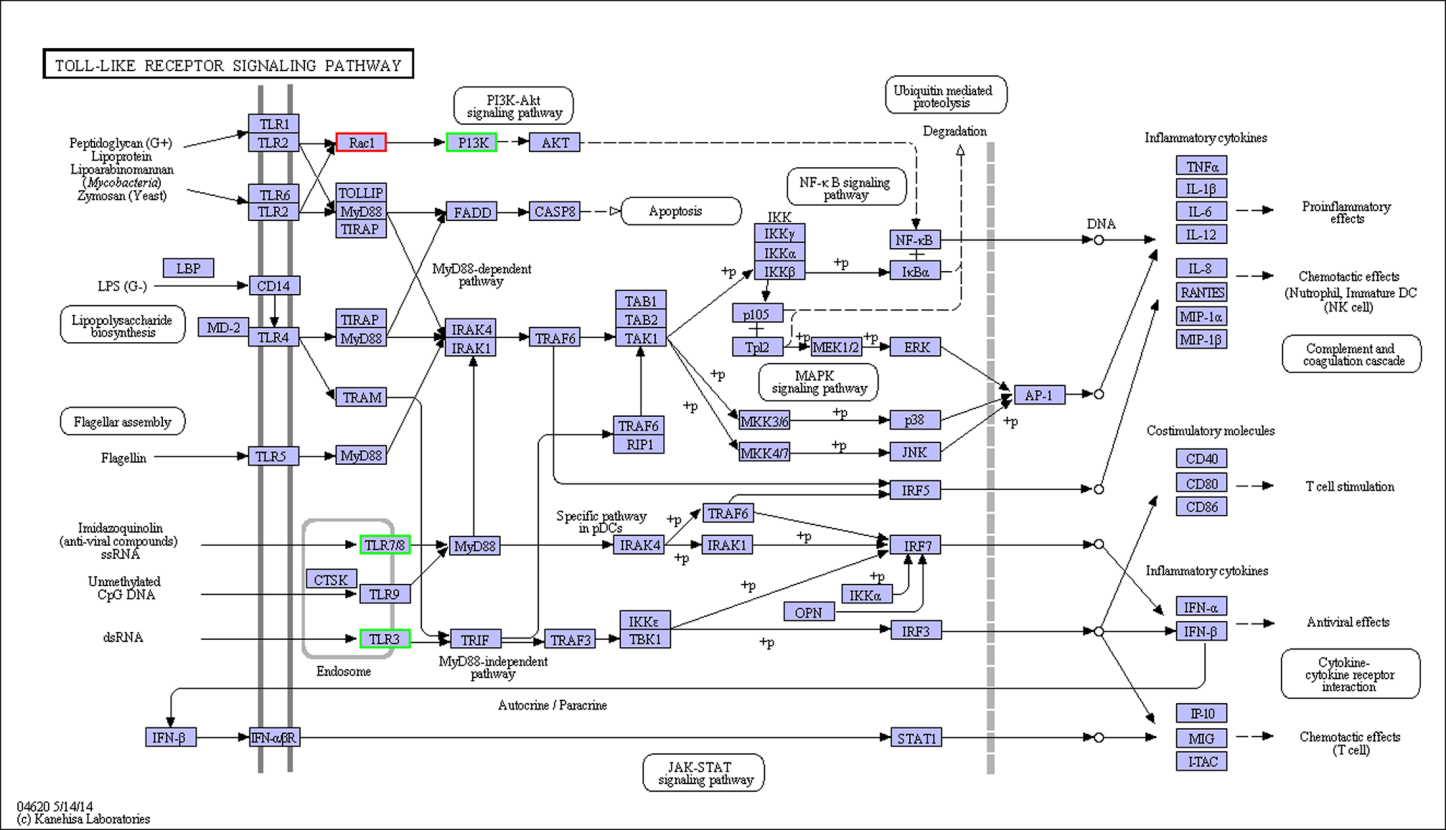
TLR signaling pathway.

**Fig 6:**
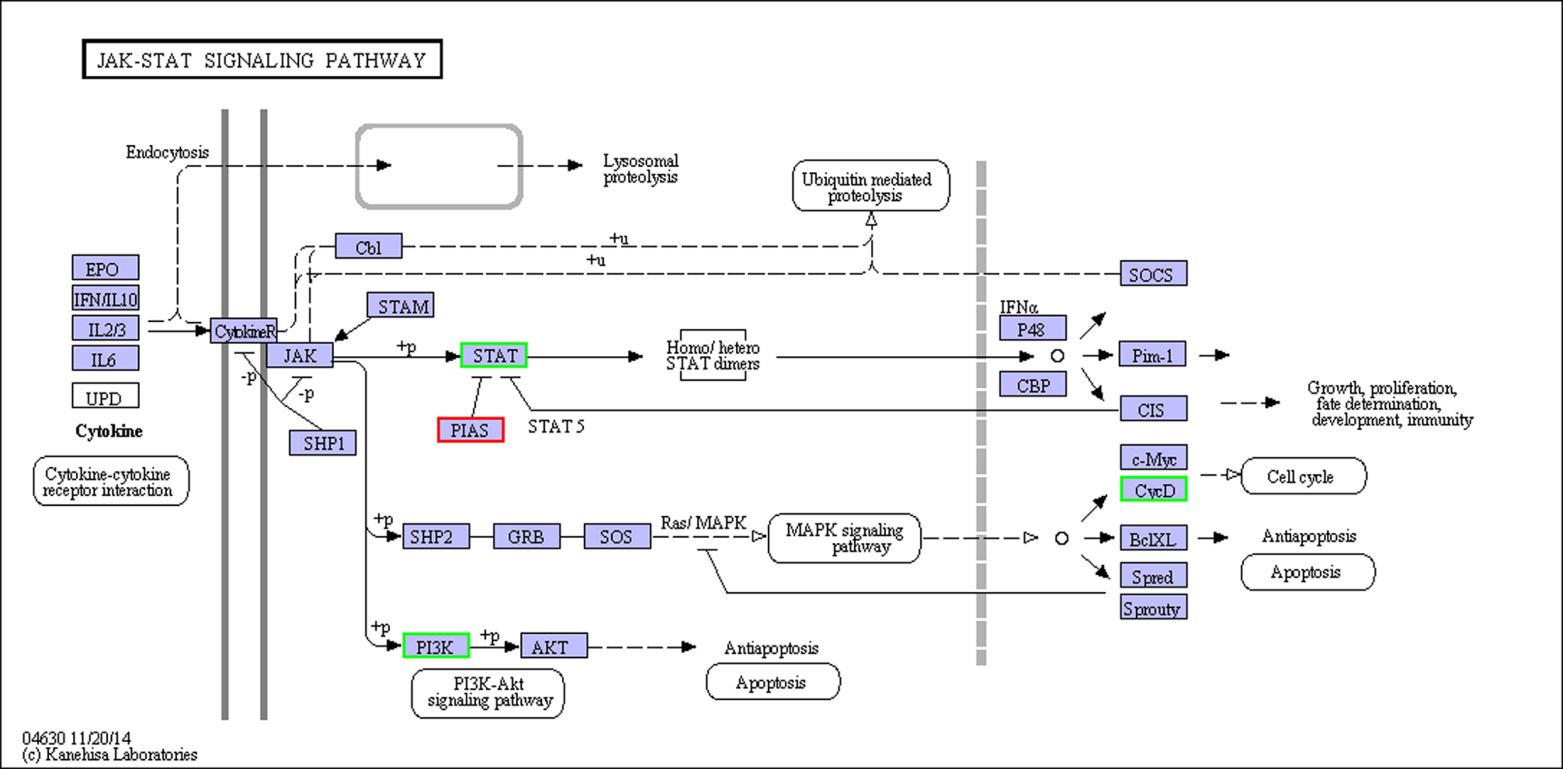
JAK—STAT signaling pathway.

We found that 1157, 1238, 412, and 3030 DEGs were significantly altered between the FO vs. SO, FO vs. LO, FO vs. FSO, and FO vs. FLO groups, respectively. Cluster analysis showed that the DEGs in each group could be divided into five clusters according to their expression levels (S4 Fig). 120 (75.68%) were in subcluster 1, where the expression of most unigenes was lower in the FO, LO, and FLO groups (S5 Fig). Cluster analysis also revealed the same expression pattern for DEGs in the FO and FSO groups; DEGs in the LO group were clustered together with that in the FLO and SO groups, but were closer to that of the FLO group. Compared with the FO group, 648, 524, 179, and 1301 genes were upregulated and 509, 714, 233, and 1729 genes were downregulated in the SO, LO, FSO, and FLO groups, respectively. To understand the DEGs further, the DEGs were annotated in the GO and KEGG databases. GO annotation showed that the expression of the genes for growth, immune system processes, enzyme regulator activity, and nutrient reservoir activity in the SO group were all upregulated compared with that of the FO group (S6 Fig). However, the expression of the genes involved in growth and immune system processes were unaltered in the LO group; the expression of the genes for the reproduction and reproductive processes were all upregulated (S7 Fig). In the FSO group, the relative expression of the genes for antioxidant activity was upregulated (S8 Fig). KEGG annotation showed that the significantly altered DEGs in the SO, LO, FSO, and FLO groups were assigned to 181, 180, 58, and 223 KEGG pathways, respectively, including that for fat digestion and absorption (Fig 1), fatty acid degradation (Fig 2), fatty acid metabolism (Fig 3), fatty acid biosynthesis (Fig 4), unsaturated fatty acid biosynthesis (S9 Fig), and several other lipid metabolism pathways. The expression of pancreatic lipase (PL) in fat digestion and absorption was significantly downregulated in the LO and FLO groups vs. the FO group. In fatty acid degradation, the expression of long-chain acyl-CoA synthetases (ACSLs) was significantly lower in the LO and FLO groups than in the FO group, but carnitine palmitoyltransferase I (CPTI) expression was upregulated. The expression of acetyl-CoA carboxylase (ACC) and fatty acid synthase (FAS) were downregulated in the LO and FLO groups as compared to the FO group. Two pathways related to immunity were downregulated in the crabs fed with vegetable oils: Toll-like receptors (TLRs) and signal transducers and activators of transcription (STAT), which play an important role in the TLR signaling pathway (Fig 5) and Janus activated kinase—STAT (JAK—STAT) signaling pathway (Fig 6), respectively.

### qRT-PCR validation of RNA-Seq

**Fig 7:**
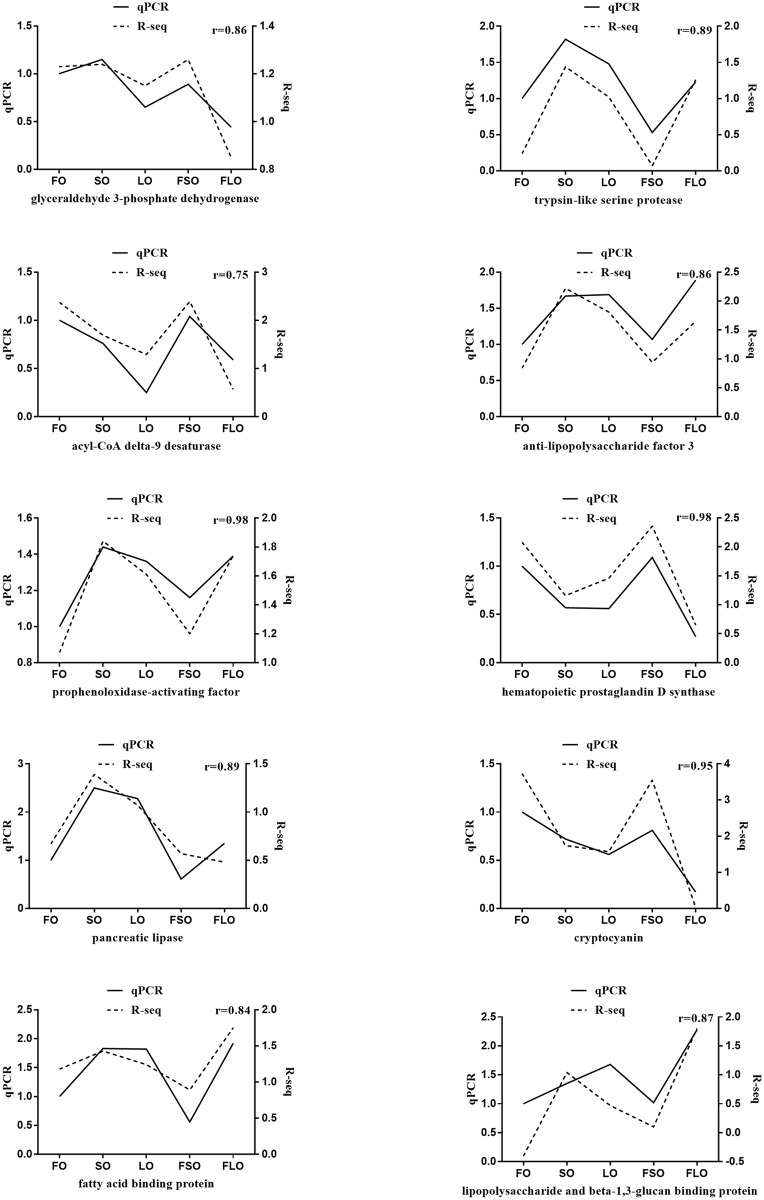
qPCR validation of RNA-Seq.

To verify the RNA-Seq results, 10 randomly selected genes in the same hepatopancreas RNA samples were analyzed by qRT-PCR. The RNA-Seq and qRT-PCR results are compared in Fig 7, and confirmed the reliability of RNA-Seq.

## Discussion

In previous years, transcriptome analysis has been extensively applied in biological studies. In crustaceans, the hepatopancreas is a major lipid storage and metabolism organ that has the same functions as adipose tissue and the liver in vertebrates [32, 33]. The hepatopancreas is also responsible for the biosynthesis of some hormones; therefore, it is the ideal organ for studying transcriptome changes following feeding with different lipid source diets [34]. In this study, we analyzed the effects of different dietary lipid resources on the *E*. *sinensis* hepatopancreas transcriptome. The dietary lipid sources had obvious effects on lipid digestion, absorption, and metabolism in *E*. *sinensis*.

Along with proteins, lipids are the major organic constituents of aquatic animals, playing an important role in the life histories and physiology aquatic animals. The major role of lipids in fish is providing energy for growth and movement [35]. Fat digestion and absorption is the first step in utilizing lipids, and determines lipids utilization in fish. Only hydrolyzed fat can be absorbed as free fatty acids and glycerides and then be reconstituted to triglycerides in the mucosal cells of the small intestine [36]. PL, which can catalyze triacylglycerol hydrolysis, is considered the most important enzyme in fat digestion and absorption. Nutritional status and hormones regulate PL expression in humans and mice [37, 38]. In the present study, crabs fed with vegetable oils had significantly lower mRNA levels of PL than those fed FO. In male weanling Sprague-Dawley rats, PL mRNA levels were lower in rats fed trioctanoate/tridecanoate (medium-chain triglycerides) than in those fed safflower oils, but were not significantly different among the diets [39]. From the results, we could conclude that comparing with long chain and high unsaturated fatty acid, short carbon chain length and low degree of desaturation of fatty acid could inhibite the mRNA levels of PL. Although the low mRNA levels of PL were found in vegetable oil, it was interesting that the hepatosomatic index in the crabs fed with vegetable oil were higher than crabs fed with FO (data were not shown). We speculated that the crabs fed with vegetable oil might have higher PL activity to increase the utilization of dietary lipid. And it had been reported that the expression and activity of PL in rat were not in synchronization when fed with different lipid diets [39], which might provide an evidence to our conclusion.

The dietary lipids significantly changed two key enzymes in β-oxidation. Compared with FO, ACSLs in vegetable oil group were downregulated, but CPTI was significantly upregulated due to the use of fatty acids in β-oxidation. ACSL and CPTI had important roles in the esterification of fatty acids and the entry of acyl moieties into the mitochondrion for β-oxidation, respectively [40–44]. ACSL1 and ACSL4 were annotated in the present study. ACSL1 expression was unchanged in the groups; ACSL4 expression was downregulated in the LO vs. FO and FLO vs. FO groups, but was unchanged in the SO vs. FO and FSO vs. FO groups. It had been reported that ACSL4 has more specific substrate preferences and has a clear preference for PUFAs, such as ARA and EPA [45, 46]. In the present study, the ARA and EPA content in the FO group were almost twice that in the FLO group. The high content ARA and EPA in FO might provide an evidence to the high ACSL4 mRNA, because more ACSL4 was needed to activate ARA and EPA. However, little is known about the reason why the ACSL4 mRNA was not changed in the SO and FSO vs. FO. CPTI is located in the mitochondrial outer membrane, and can convert fatty acid-CoAs into fatty acid carnitines [47]. In the present study, CPT1 mRNA was significantly increased in the FLO group rather than the FO group. There was study indicated that the fatty acids could have an effect on CPTI mRNA, where long-chain fatty acids such as 16:0, 18:1n-9, 18:2n-6, 20:5n-3, and 22:6n-3 can significantly increase CPTI mRNA as compared to medium-chain fatty acids (8:0 and 10:0), and the effects of 20:5n-3 and 22:6n-3 are more significant than that of 16:0, 18:1n-9, and 18:2n-6 [48]. The result above was contrary to the present study, we speculate that this is due to the different species between rat and *E*. *sinensis*. There are high content of EPA and DHA in the hepatopancreas of *E*. *sinensis*. Dietary EPA and DHA in FO might mainly be stocked in hepatopancreas rather than provide energy by β-oxidation, thereby decreasing the expression of CPTI.

The de novo synthesis of fatty acid was also significantly changed by the dietary lipid. Fatty acid synthesis involves two steps: ACC and FAS catalyze the first, and then the fatty acids synthesized by ACC and FAS are further elongated and desaturated into long-chain unsaturated fatty acids [49]. The reaction begins with the synthesis of malonyl-CoA from acetyl-CoA, catalyzed by ACC. Then, sequential Claisen condensation reactions catalyzed by FAS take place with acetyl-CoA and malonyl-CoA [50]. It had been reported that the expression of FAS and ACC in CaCo-2 cells could have a close relationship to the fatty acids [51]. In the present study, comparing with the FO group, FAS was downregulated in the SO, LO, and FLO groups and ACC was downregulated in the FLO group. The greatest difference between the FO, SO, LO, and FLO diets is the fatty acid composition. However, comparing with vegetable oil, FO could significantly promote the expression of FAS. we speculated that this was due to the high content of 14:0 in the diet of FO, which was the substrate of FAS.

*E*. *sinensis* is famous for the high PUFA content in the hepatopancreas. PUFA biosynthesis is complex and involves sequential desaturation and elongation catalyzed by fatty acyl elongase (ELOVL) and desaturase (FAD) [52, 53]. We have investigated the capacity of PUFA biosynthesis in *E*. *sinensis*, and have identified many enzymes participating in PUFA biosynthesis, such as fatty acyl Δ6-desaturase, fatty acyl Δ9-desaturase, and *ELOVL6* [54–56]. *ELOVL2* and *ELOVL5* were annotated in the present study. *ELOVL2* and *ELOVL5* are widely distributed in mammals, bony fish, and other vertebrates [57–59]. Molecular characterization of *ELOVL2* and *ELOVL5* has shown that they are related to the biosynthesis of long-chain fatty acids such as EPA and DHA [60]. The expression of *ELOVL2* and *ELOVL5* in *E*. *sinensis* provides compelling evidence for the high EPA and DHA content in *E*. *sinensis* hepatopancreas. Many studies have indicated that different lipids can regulate *ELOVL* expression. Substituting FO with vegetable oils significantly increased *ELOVL* expression [61–63]. However, in this study, *ELOVL2* and *ELOVL5* expression was unaltered following the replacement of FO by SO and LO. There is not much preceding information on the difference between our study and others. Further studies should be performed to obtain the full-length cDNA of *ELOVL2* and *ELOVL5* in *E*. *sinensis* and to investigate the effects of dietary fatty acids on *ELOVL*.

But in the present study, fatty acyl Δ9-desaturase expression was significantly altered in the SO, LO, and FLO groups. Fatty acyl Δ9-desaturase is the rate-limiting enzyme in monounsaturated fatty acid biosynthesis, and can introduce a double bond in palmitoyl-CoA (16:0) and stearoyl-CoA (18:0) [64]. Guo and colleagues were the first to isolate fatty acyl Δ9-desaturase from *E*. *sinensis* [55], and it has been characterized in BL21(DE3)pLysS, fatty acyl Δ9-desaturase in *E*. *sinensis* had an activity in the desaturation of C18:0 [65]. As the high content of 18:1n-9 in the vegetable oil, the fatty acyl Δ9-desaturase expression in FO was significantly lower than vegetable oil. We speculate that the high content of 18:1n-9 in the vegetable oil are the products of the reaction in which fatty acyl Δ9-desaturase participates, thereby inhibiting fatty acyl Δ9-desaturase expression in *E*. *sinensis*.

Many studies have indicated that replacing FO with vegetable oils did not affect growth in aquatic animals, but that immunity could be reduced significantly [6, 9]. In the present study, replacing FO with vegetable oils had significant effects on the TLR and JAK—STAT signaling pathways and many other pathways related to immunity. The TLR signaling pathway is the innate immune system of invertebrates for sensing pathogenic microorganism invasion. TLRs play a crucial role in this system, which can recognize specific microbial components [66]. Currently, there are 12 known TLRs (TLR1–TLR12) [67]. TLRs are important pattern recognition receptors (PRRs), which have different ligands. TLR3 is required for the recognition of double-stranded RNAs (dsRNAs) [68]; single-stranded RNAs (ssRNAs) are mainly recognized by TLR7 and TLR8 [69, 70]. In the present study, TLR3, TLR7, and TLR8 were significantly downregulated during LO replacement of FO, which might decrease virus recognition and the immune response further. STATs are a family that can be activated by JAK family members, and regulate downstream pathways such as the growth and differentiation of immune cells [71, 72]. The JAK—STAT signaling pathway plays an important role in the immunologic defense of crustaceans [73, 74]. Compared with FO, STAT was significantly downregulated in the crabs fed with vegetable oils, and the downregulated protein inhibitors of activated STAT is an effective inhibitor of STAT. The change in STATs could have a significant effect on the immune system of *E*. *sinensis*.

## Conclusions

The effects of dietary lipid resources on the Chinese mitten crab were analyzed with transcriptome analysis, which showed that dietary lipids had obvious effects on the lipid metabolism in the hepatopancreas of the crabs. Replacing FO with vegetable oils significantly altered fat digestion and absorption, fatty acid metabolism, fatty acid degradation, fatty acid biosynthesis, unsaturated fatty acid biosynthesis, and many other lipid metabolism pathways. Compared with FO, the increasing addition of SO and LO in the diets of the crabs might decrease the digestion and absorption of dietary lipids, fatty acids biosynthesis, and virus immunologic defense, and increase β-oxidation by altering the expression of the genes for PL, ACSLs, CPTI, ACC, FAS, fatty acyl Δ9-desaturase, TLRs, STAT, and other relevant genes. However, the details of the effects of dietary lipids on Chinese mitten crab are still unclear; future studies should use the *E*. *sinensis* genomic sequence to improve the transcriptome. Moreover, the present study was conducted at transcriptional level; protein expression should also be analyzed to further understand the lipid metabolism of Chinese mitten crabs fed different lipid diets.

## Supporting information

S1 Fig Unigene GO annotation (level 2). (TIF)

S2 Fig Unigene COG annotation. (TIF)

S3 Fig Unigene KEGG pathway annotation. (TIF)

S4 Fig DEG cluster analysis. (TIF)

S5 Fig Trend lines for five subclusters in cluster analysis. (TIF)

S6 Fig GO annotation of DEGs in FO vs. SO. (TIF)

S7 Fig GO annotation of DEGs in FO vs. LO. (TIF)

S8 Fig GO annotation of DEGs in FO vs. FSO. (TIF)

S9 Fig Biosynthesis of unsaturated fatty acid pathway. (TIF)
