# Supplementary figures and images for: Effects of dietary lipids on the hepatopancreas transcriptome of Chinese mitten crab (Eriocheir sinensis)
Source: PLoS One. 2017 Jul 28;12(7):e0182087. doi: 10.1371/journal.pone.0182087 (PMC5533325; doi:10.1371/journal.pone.0182087)

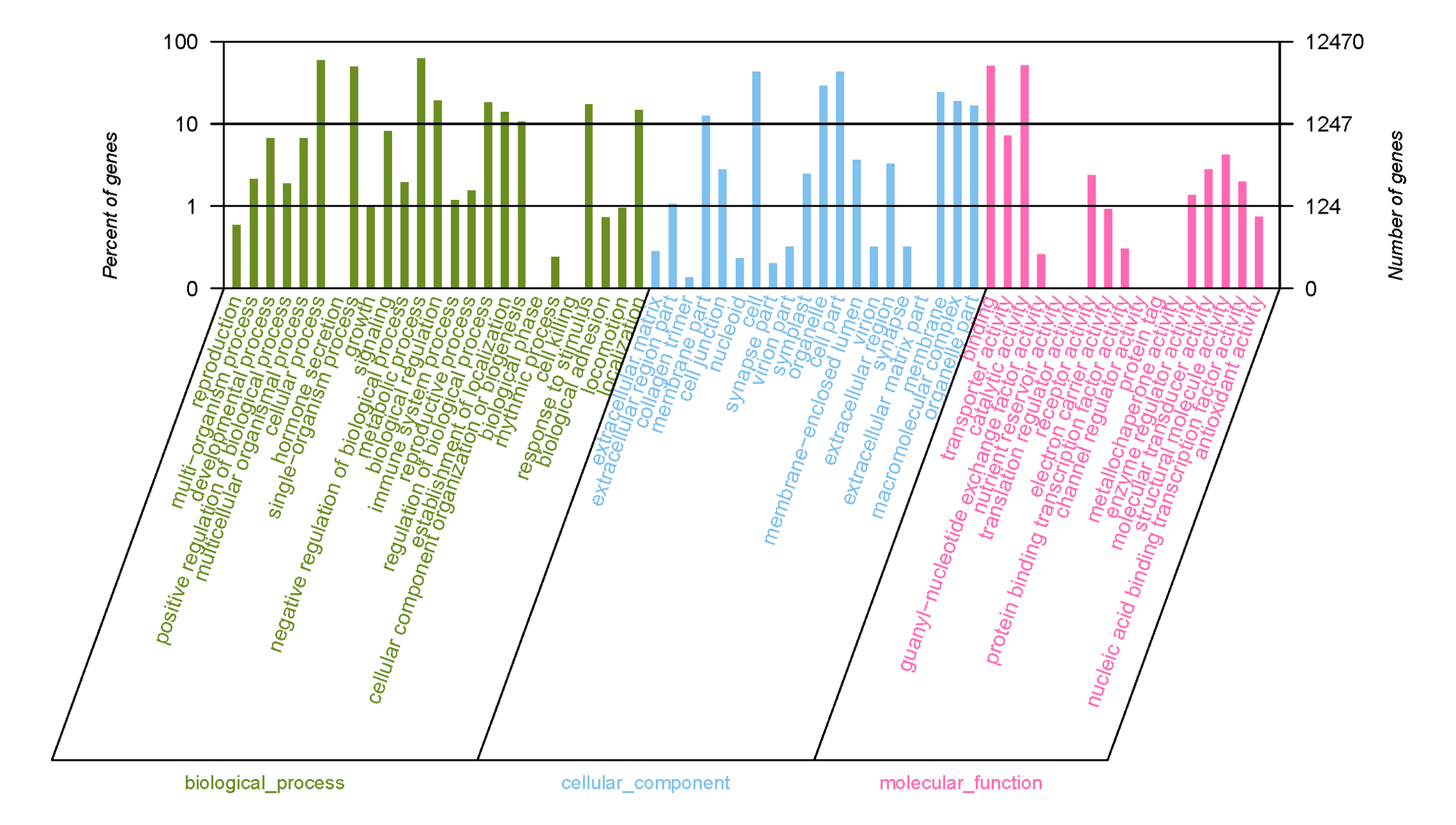

Supplement: S1 Fig — (TIF) [file pone.0182087.s001.tif]

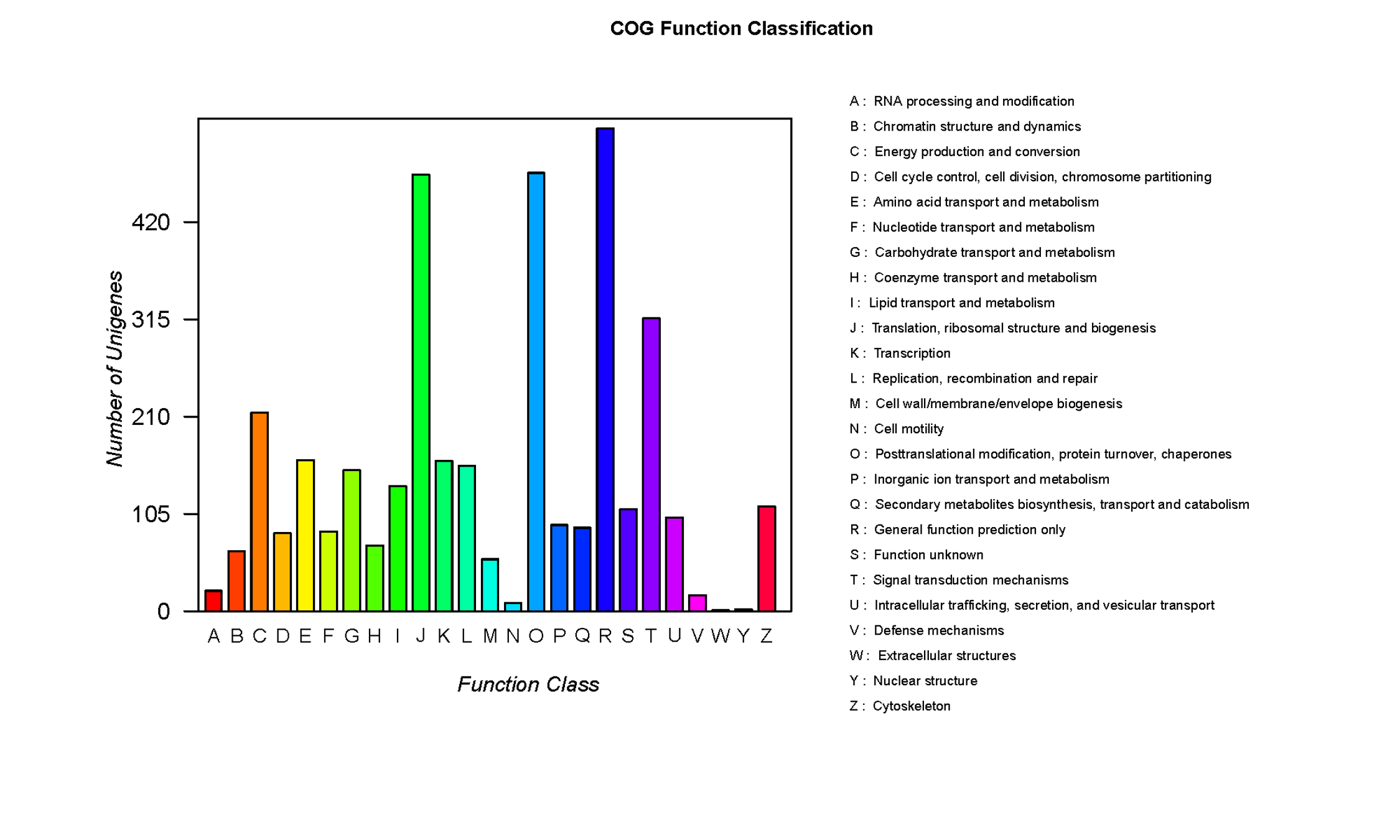

Supplement: S2 Fig — (TIF) [file pone.0182087.s002.tif]

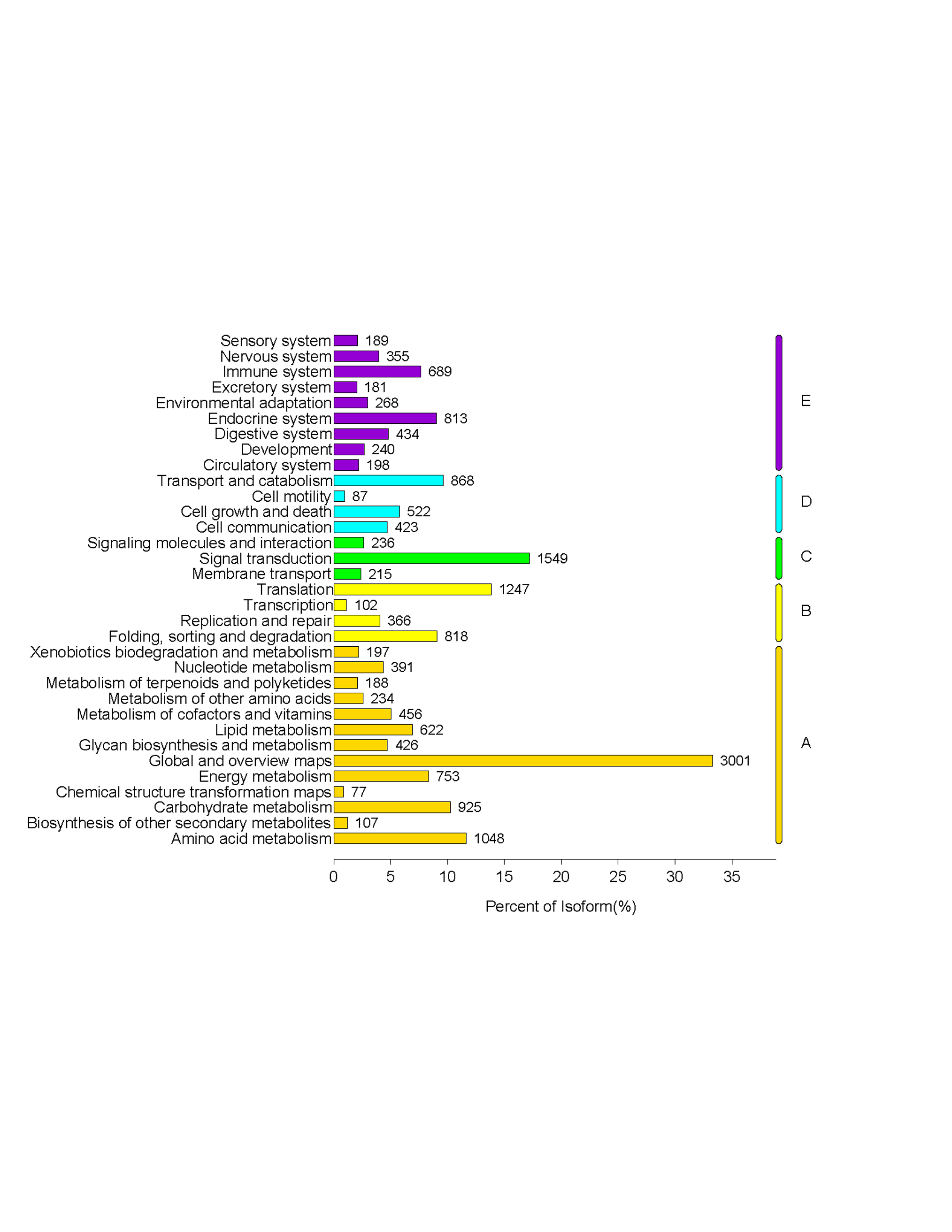

Supplement: S3 Fig — (TIF) [file pone.0182087.s003.tif]

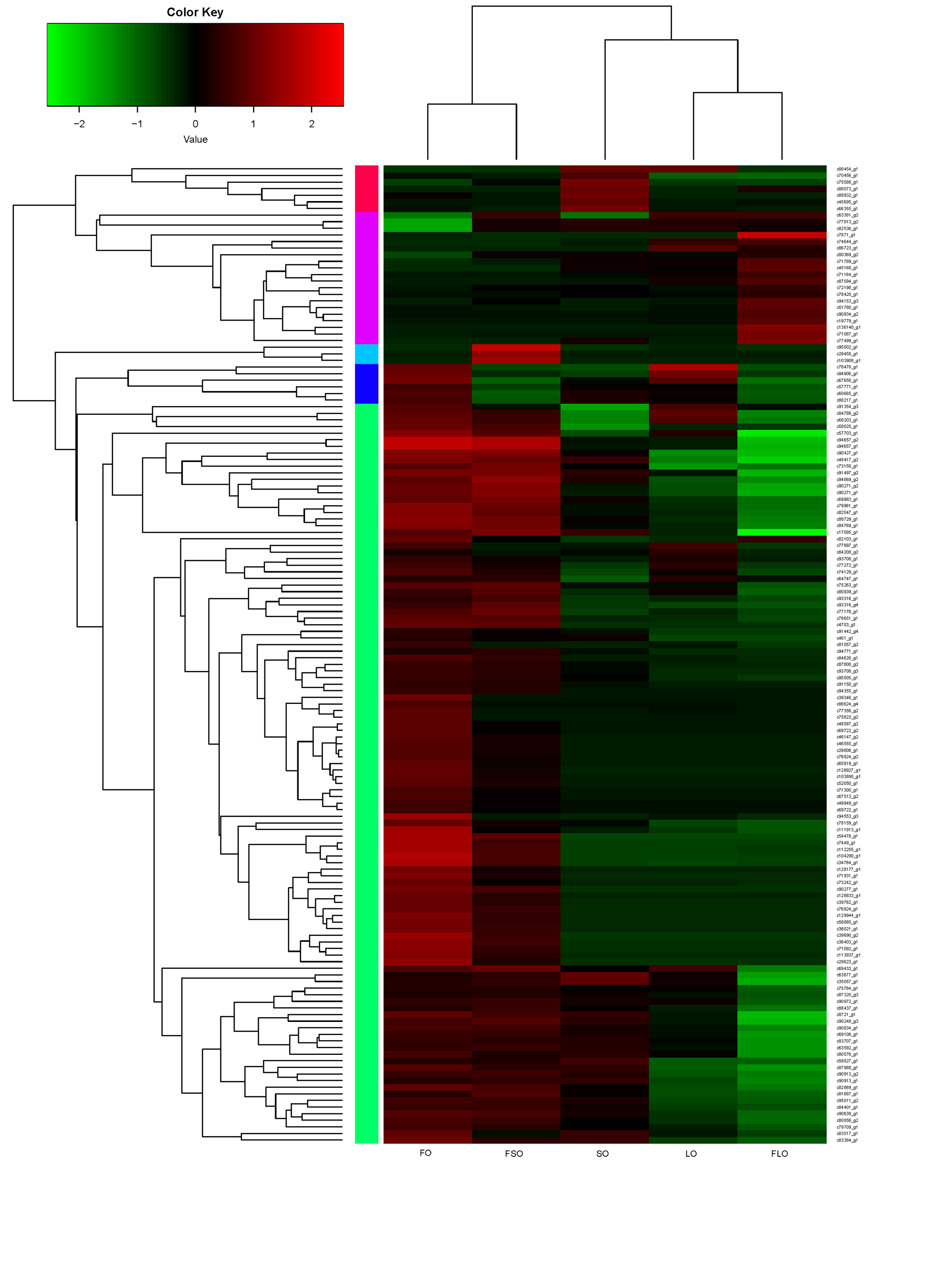

Supplement: S4 Fig — (TIF) [file pone.0182087.s004.tif]

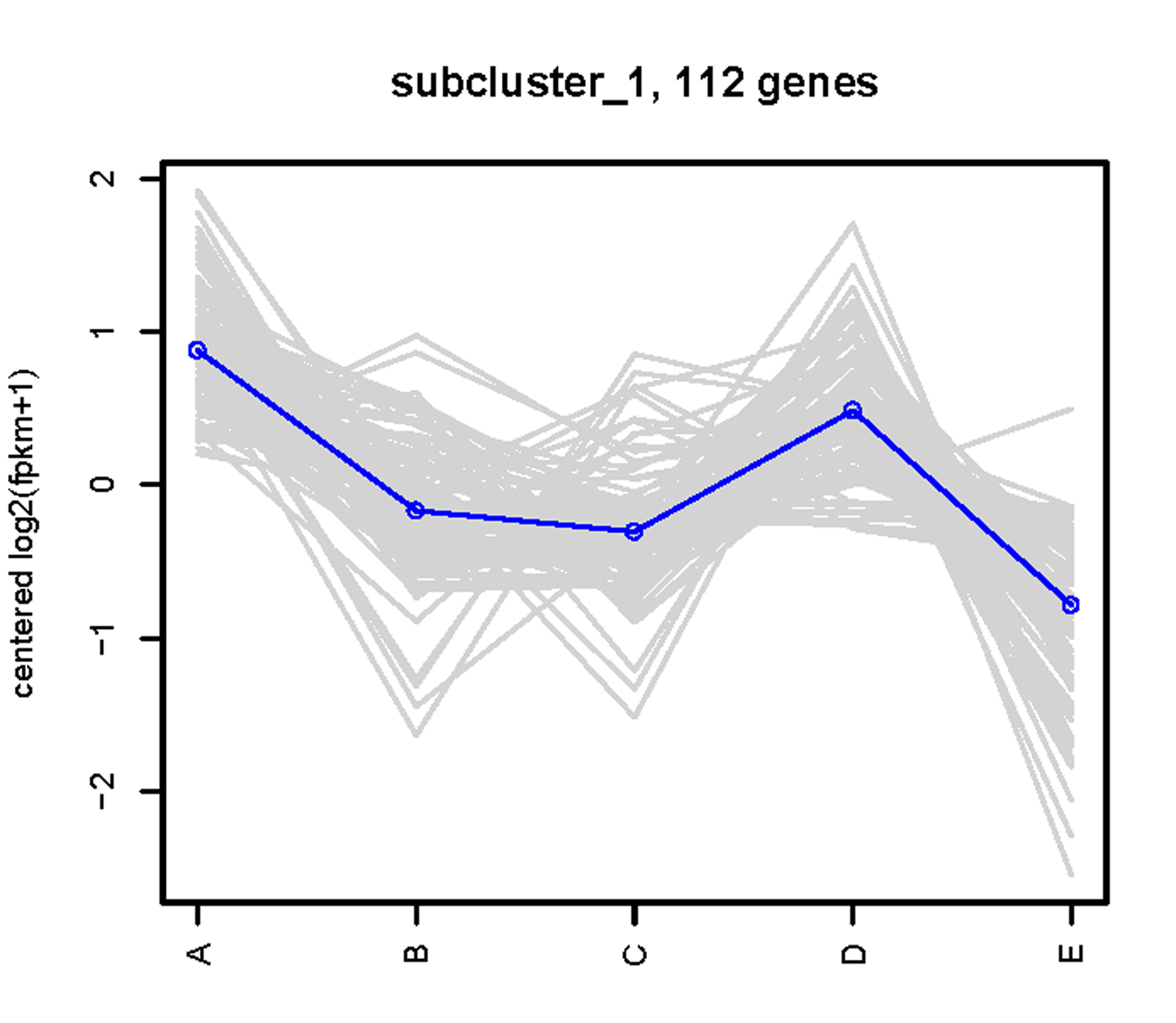

Supplement: S5 Fig — (TIF) [file pone.0182087.s005.tif]

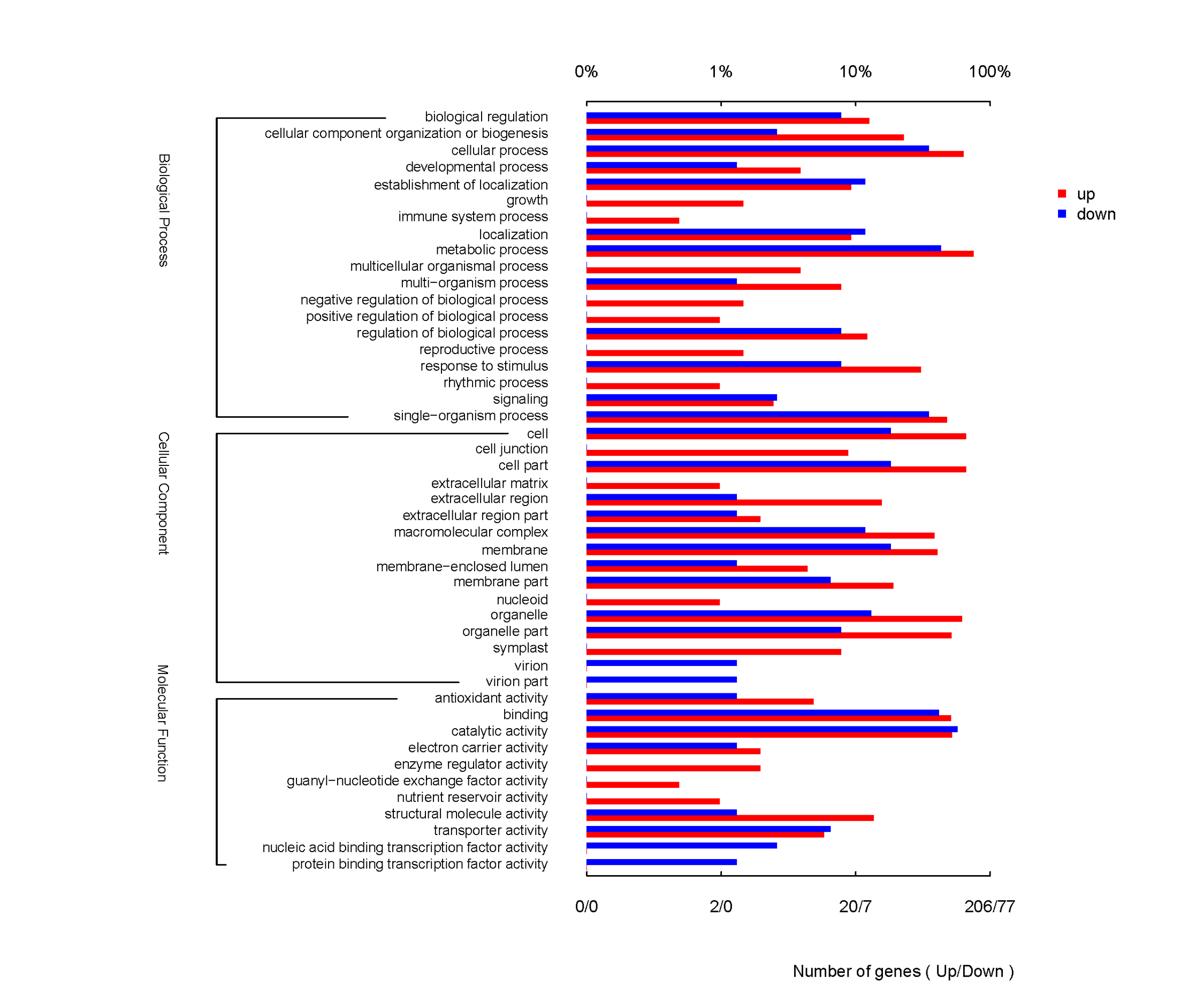

Supplement: S6 Fig — (TIF) [file pone.0182087.s006.tif]

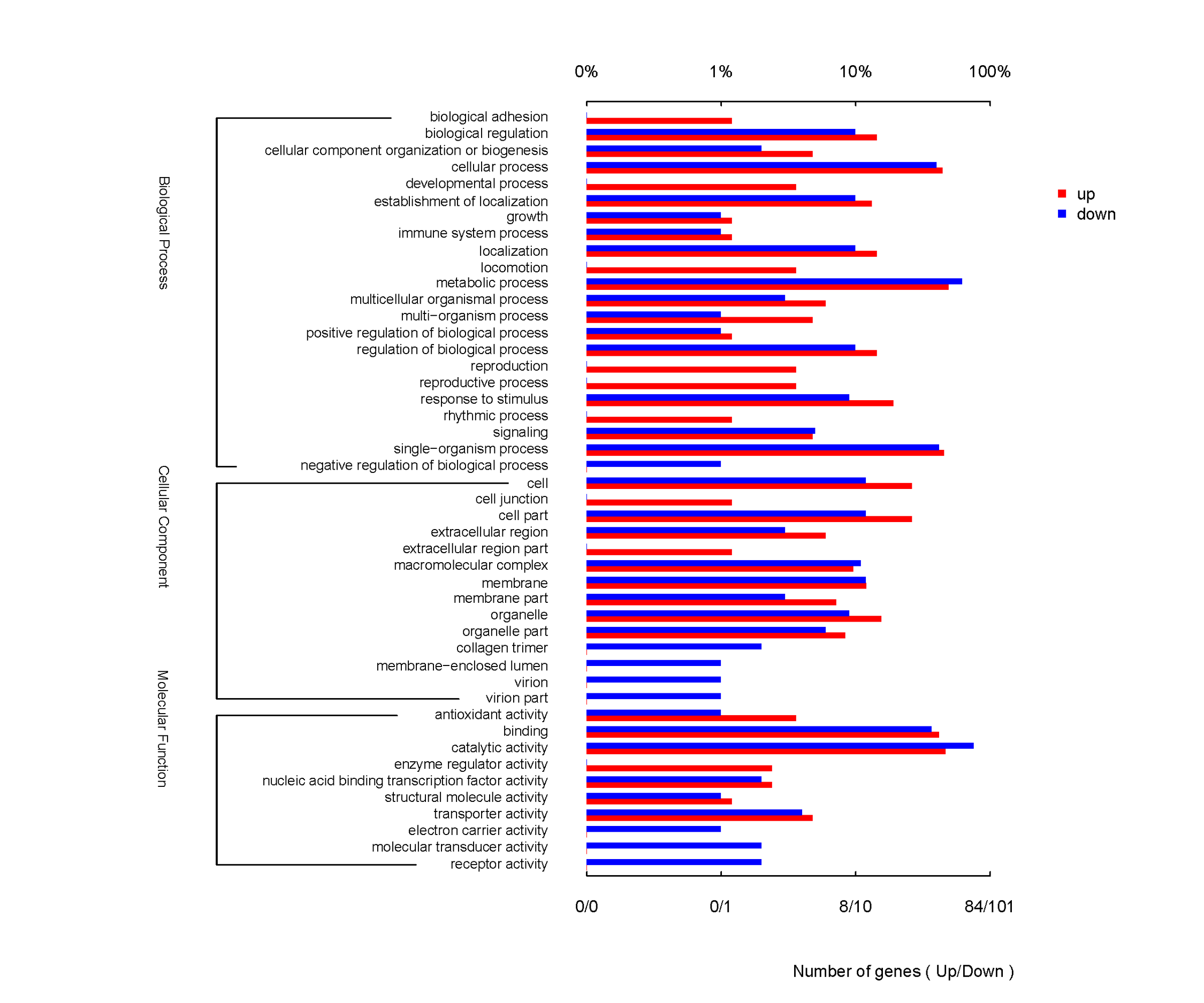

Supplement: S7 Fig — (TIF) [file pone.0182087.s007.tif]

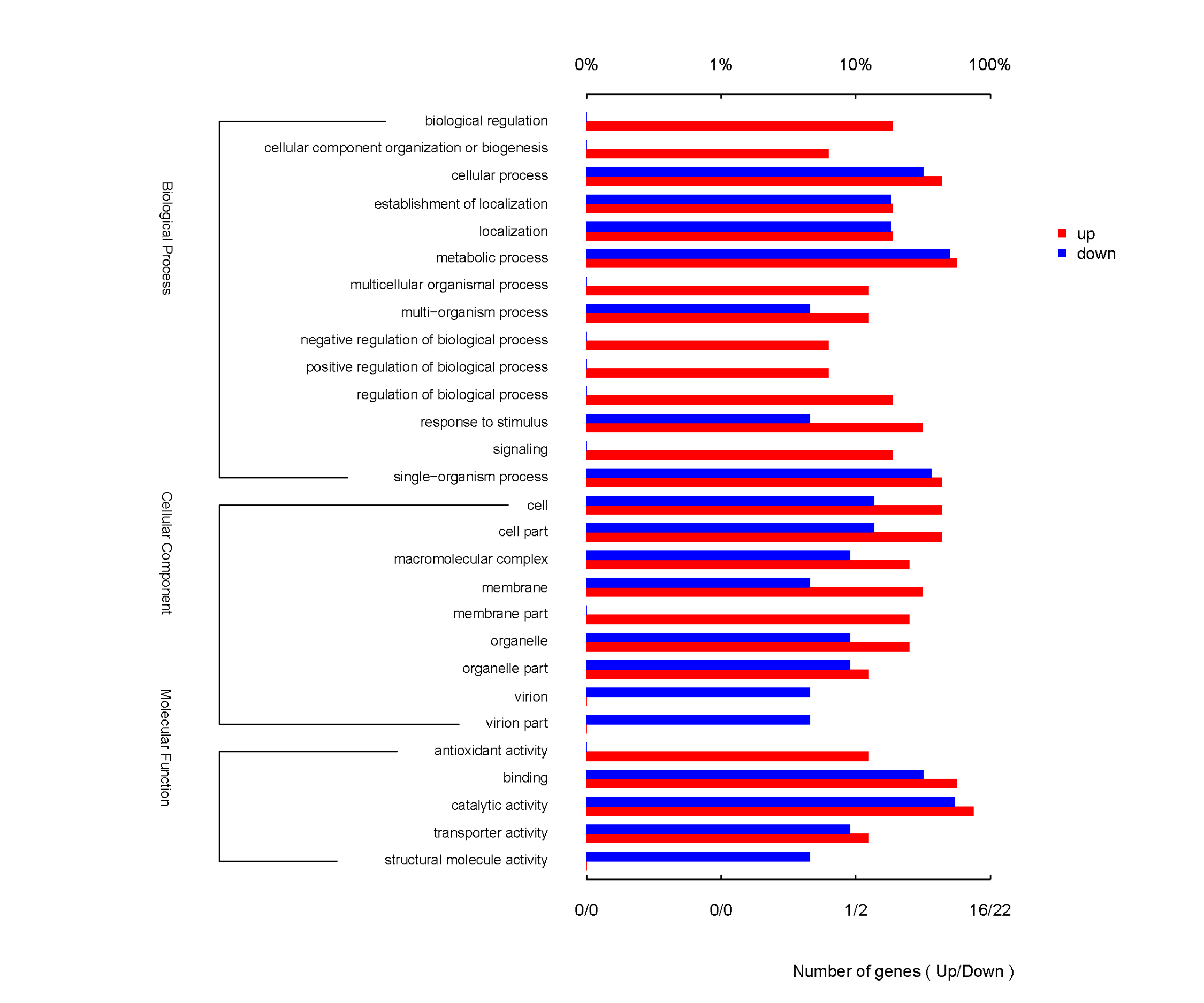

Supplement: S8 Fig — (TIF) [file pone.0182087.s008.tif]

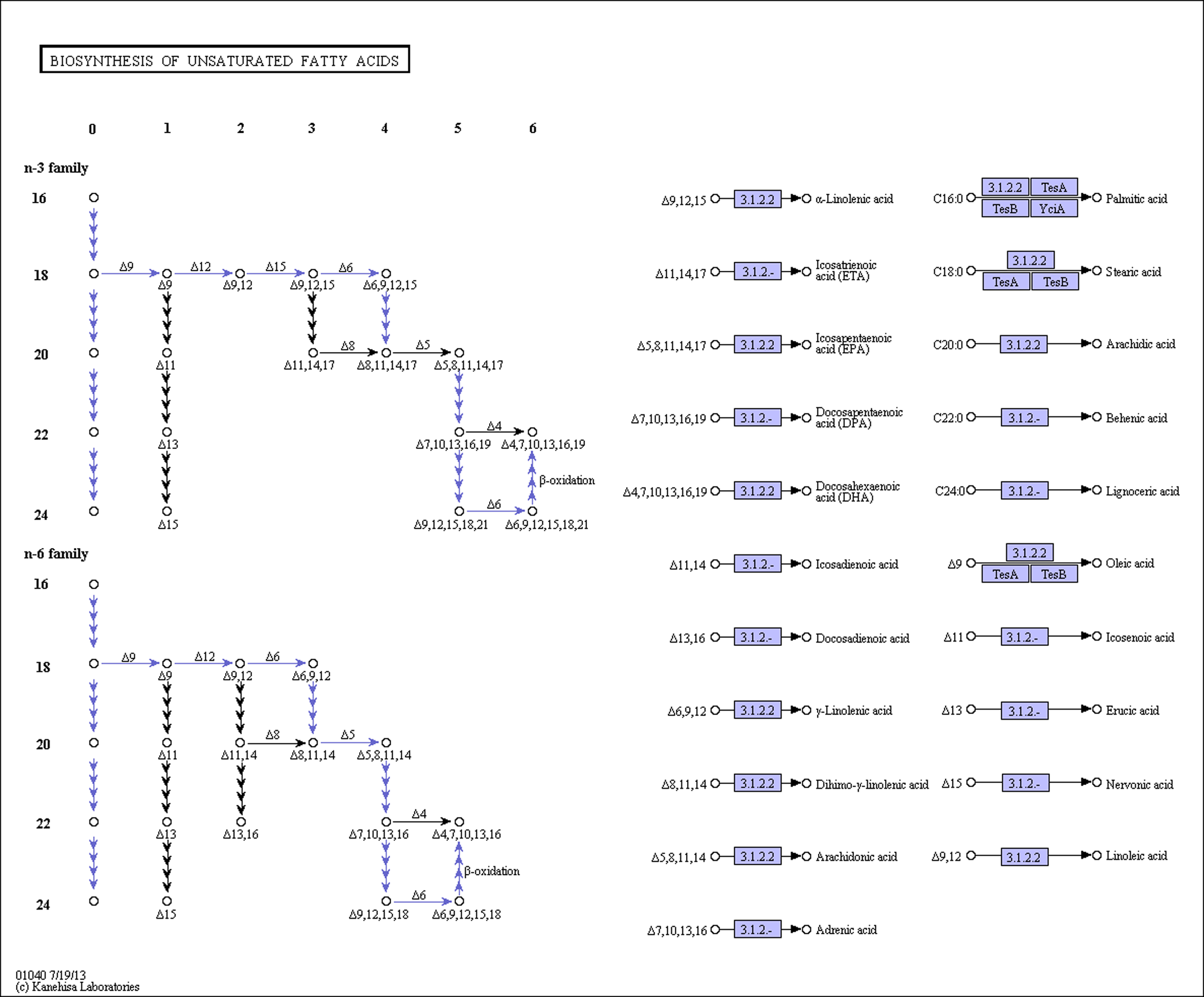

Supplement: S9 Fig — (TIF) [file pone.0182087.s009.tif]
